# Precision medicine from a citizen perspective: a survey of public attitudes towards pharmacogenomics in Flanders

**DOI:** 10.1186/s12920-022-01308-7

**Published:** 2022-09-12

**Authors:** Ahmed Edris, Evi Callier, Lies Lahousse

**Affiliations:** Department of Bioanalysis, Faculty of Pharmaceutical Sciences, Ghent University, Ottergemsesteenweg 460, 9000 Ghent, Belgium

**Keywords:** Pharmacogenetics, Patient perspective, Research engagement, Survey, Public attitude

## Abstract

**Background:**

Personalized medicine is an emerging field, aiming to improve the safety and efficacy of pharmacotherapy. The field’s implementation in clinical care is steadily increasing. Pharmacogenomics are one example of personalized approaches in the clinic and direct-to-consumer (DTC) pharmacogenomic tests have become publicly available. We aimed to assess public opinion on pharmacogenomic research and testing to foster integration within Belgian health care.

**Methods:**

A cross-sectional survey was created and disseminated online, focusing on the citizen perspective. Participants’ willingness to engage in pharmacogenomic research was the primary outcome. In addition, their awareness, understanding, expectations and overall acceptance towards pharmacogenomic testing was investigated.

**Results:**

A total of 156 participants (54.5% aged between 18 and 30 years, 45.5% > 30 years; 73.1% females) completed the survey. Half ever experienced side effects (46.2%) and treatment failure (52.6%). Up to 45.5% (n = 71) were willing to participate in pharmacogenomics research, and the majority (78.8%) were convinced that pharmacogenomic tests could help doctors to prescribe them the right medications. Additionally, 76.3% (n = 118) supported a partial reimbursement of pharmacogenomics tests. A minority (5.1%, n = 8) of participants showed interest in DTC tests, and 15.4% (n = 24) expressed privacy concerns regarding pharmacogenomics testing. Participants preferred their healthcare professionals’ to perform the test and access their data, but refused commercial providers.

**Conclusion:**

Overall, participants showed a positive attitude towards precision medicine and pharmacogenomics research. Our findings may help guiding future pharmacogenomic implementation initiatives to optimize drug use by using pharmacogenomic information integrated within health care.

**Supplementary Information:**

The online version contains supplementary material available at 10.1186/s12920-022-01308-7.

## Introduction

The current approach to pharmacotherapy has several drawbacks [1], including limited drug efficacy, increased risk of drug reactions, and a longer time towards achieving therapeutic goals [2]. This results in a large economic burden as it is estimated, for example, that hospitalizations caused by Adverse Drug Reactions (ADRs) cost up to 706 million euros annually [3, 4]. Moreover, up to 5.1% of all hospital admissions in the Netherlands resulted from ADRs [5]. This can be particularly problematic in patients suffering from common chronic conditions including cardiovascular diseases, asthma, and depression [6].

Pharmacogenomics (PGx) aim to identify genetic variants influencing drug response (currently estimated to be between 25 and 80%) to optimize benefits and reduce harm of medication in clinical practice [7]. While medication response is multifactorial, specific genetic variants may have a relatively large effect on certain drug responses, especially variants affecting pharmacokinetics and pharmacodynamics [6]. Pharmacogenomic approaches in both drug and trial design may yield drugs better targeted for specific populations, therefore increasing efficacy, decreasing side effects and contributing to more personalized medicine approaches [8, 9].

The Clinical Pharmacogenomics Implementation Consortium (CPIC) and the Dutch Pharmacogenetics Working Group have both issued guidelines to clarify clinical implementation and required actions upon specific pharmacogenetic test results, and both FDA(Food and Drug Administration) and EMA (European Medicines Agency) have included pharmacogenetic information in their drug labeling [10]. However, implementation of PGx testing in routine care is still currently limited, whether worldwide or in Belgium [11]. Barriers to implementation include unclear testing requirements (pre-emptive vs reactive testing), limited healthcare professional knowledge regarding PGx, and patient concerns [12].

Patients are key stakeholders in the successful implementation of healthcare interventions, and their perspective is central to foster translational research and implementation efforts [13–17]. Lower participation may also lead to smaller sample sizes, and reduced generalizability of results to the wider population [18]. There are few studies focused on willingness to participate in pharmacogenomics research, and the main motives behind participants decisions may differ among different populations [19–21]. Although initial studies of public opinions showed positive attitudes towards PGx, several concerns have been identified: cost and insurance coverage, privacy and confidentiality issues and stress associated with incidental findings [15–17, 22]. Moreover, participants expressed mixed opinions on potential data access and authorization issues [13].

We aimed to better understand the awareness, understanding, expectations and overall acceptance of the Flemish public towards pharmacogenomic testing, aiming to better inform implementation initiatives in Belgium. This included assessing information deemed necessary by the public before undertaking tests. Furthermore, participants’ willingness to engage in pharmacogenomic research was investigated, as well as their preferences towards integration within health care or direct-to-consumer (DTC) initiatives.

## Methods

### Study design

An online cross-sectional survey was generated using Microsoft Forms. The dissemination of the survey focused on the general public, aiming to collect responses from a larger sample of people with different backgrounds. Social media was used as the primary dissemination strategy. Scivil, a Flemish organisation focusing on citizen science, agreed to publish the survey in their April 2020 newsletter. Participants had the opportunity to fill in the survey from 11 March 2020 to 28 April 2020. The survey has been approved by the UZ Ghent Ethical Committee (registration number: B670202043039). No incentives were provided, informed consent was obtained (through a required entry before accessing the survey questions) and all survey data was analyzed anonymously.

### Study questionnaire

A brief and simplified introduction surrounding pharmacogenomics was provided at the start of the survey questionnaire. The survey consisted of 25 questions—excluding the informed consent—and included the following sections: Baseline characteristics, awareness and understanding of pharmacogenomic testing, acceptance and expectations of pharmacogenomic testing, role of health care providers, and willingness to engage in pharmacogenomic research. Survey responses were imported in SPSS version 26 (SPSS Inc., Chicago, IL). The primary outcome was participants’ willingness to engage in pharmacogenomic research, calculated as the percentage of participants answering this question positively. An English version of the survey is available in the online supplement. (Additional file 1).

## Results

A total of 156 participants completed the survey (Table 1). The majority (n = 85; 54.5%), were between the ages of 18 and 30 years. While only 2 participants (1.3%) were older than 70. Approximately 3 out of every 4 participants were females (73.1%), and the majority had either a high school degree (37.2%) or a university degree (34.6%). Half (n = 78; 50.0%) were taking 1–5 drugs per day, while only 4 participants (2.6%) took more than 5 drugs daily. Seventy-two participants (46.2%) reported previous side effects caused by a medication, while 82 (52.6%) experienced treatment failure, with up to 70 (44.9%) stopping the medication due to therapeutic failure and 35.6% (n = 56) stopping the drug due to a side effect. Forty-five participants (28.8%) experienced both side-effects and drug failure.

**Table 1:** Participants characteristics

Characteristics	Total N (%) 156 (100%)
Sex	
Male	42 (26.9%)
Female	**114 (73.1%)**
Age	
18 – 30	**85 (54.5%)**
31 – 50	38 (24.4%)
51 – 70	31 (19.9%)
70 +	2 (1.3%)
Education level	
Elementary or primary degree	2 (1.3%)
High school degree	**58 (37.2%)**
College degree	38 (24.4%)
University degree	54 (34.6%)
Doctorate	4 (2.6%)
Number of average daily drugs	
0	74 (47.4%)
1 – 5	**78 (50.0%)**
5 +	4 (2.6%)
Experienced side-effects due to their drugs	
Yes	**72 (46.2%)**
No	34 (21.8%)
Not that I know of	41 (26.3%)
Not applicable	9 (5.8%)
Stopped their drug because of side-effects^1^	
Yes	56 (35.6%)
No	19 (12.2%)
Not applicable	**81 (51.9%)**
Experienced treatment failure	
Yes	**82 (52.6%)**
No	29 (18.6%)
Not that I know of	36 (23.1%)
Not applicable	9 (5.8%)
Stopped their drug due to treatment failure^1^	
Yes	70 (44.9%)
No	15 (9.6%)
Not applicable	**71 (45.5%)**

The bold entries denote the largest proportion for every question

### Understanding of pharmacogenetic testing

**Fig. 1:**
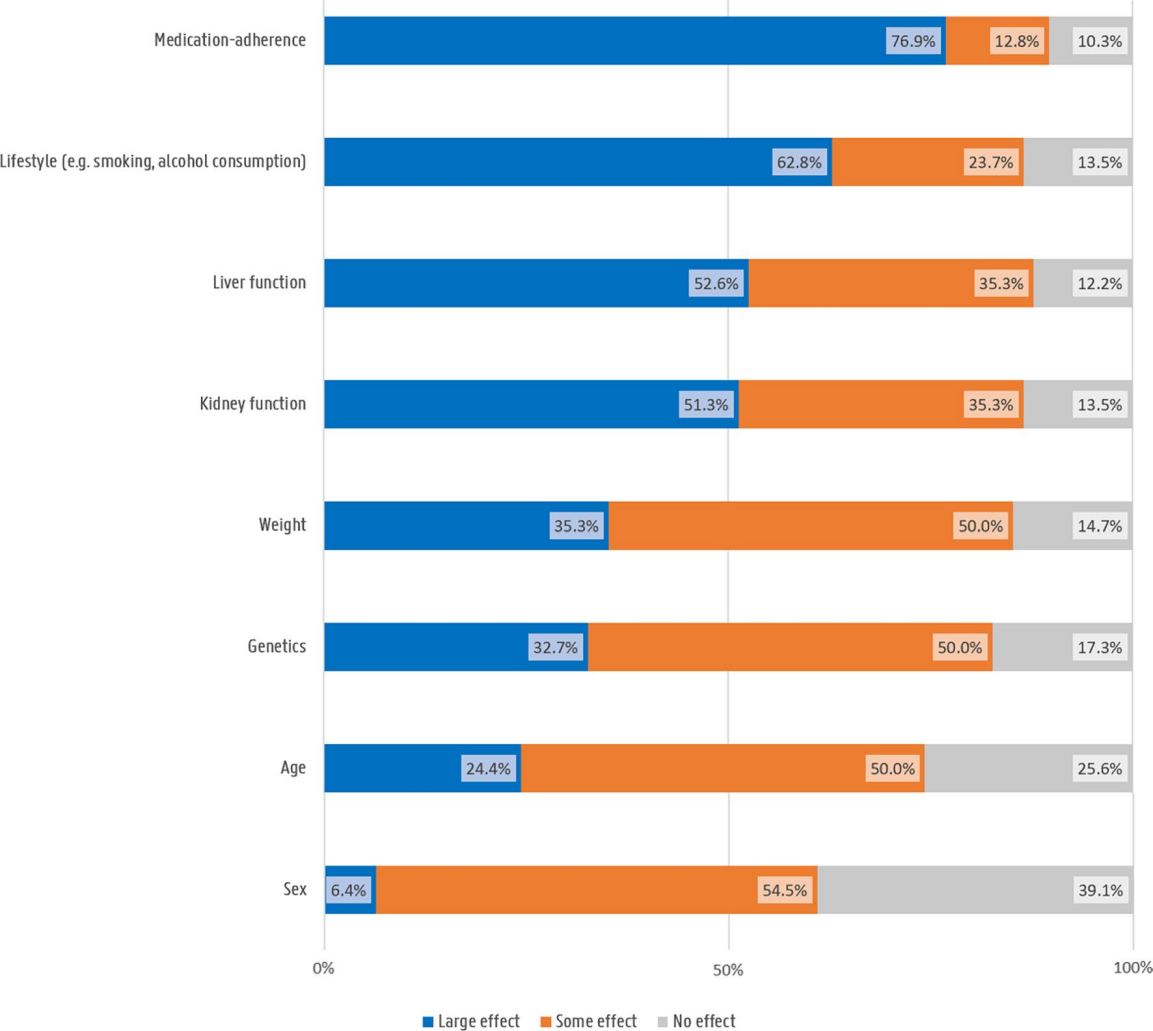
Participants ranking of factors affecting medication response

Forty-eight participants (30.8%) had heard of pharmacogenomics or personalised medicine before the survey. Most participants (n = 123; 78.8%) believed that genetic variation data could help physicians to prescribe the right medication. Most participants described medication-adherence, lifestyle, liver and kidney function as having a ‘large effect’ on the safety or the efficacy of drugs. Other factors (weight, genetics, age, sex) were mostly ranked as having ‘some effect’. (Fig. 1) Thirty-five participants (22.4%) had heard of DTC genetic testing. None of them had ordered such a test. However, 8 participants (5.1%) would consider ordering a DTC genetic test in the future.

### Acceptance and expectations of pharmacogenomic testing

**Fig. 2:**
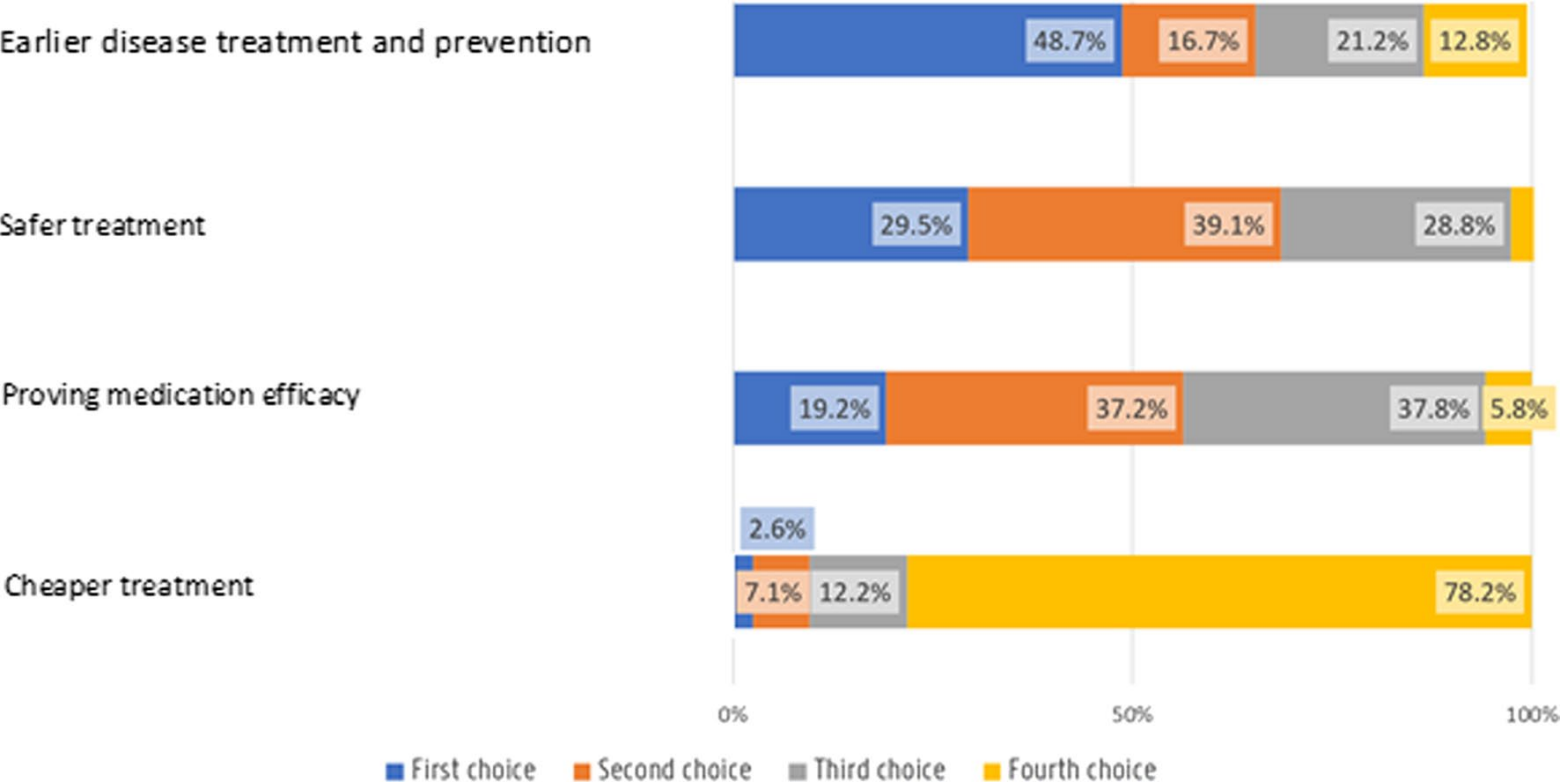
Preferred purpose of a pharmacogenomic test according to participants

Almost half of participants (n = 76; 48.7%) chose earlier disease treatment and prevention as the most important expected benefit of pharmacogenomics testing. Conversely, proving that a cheaper drug was as effective as an expensive one was the least important benefit according to survey participants as a majority (n = 122; 78.2%) ranked the identification of a cheaper drug as their lowest priority. Proving the safety and efficacy of a drug were mostly ranked as second and third choices, respectively. (Fig. 2).

Table 2 provides all responses to statements regarding pharmacogenomic tests. Main consensus was for pharmacogenomic tests to be an important aid during health care research (93.6% at least agreed) and to improve health care (87.2% at least agreed). Moreover, most participants agreed that they would feel more at ease when taking a drug that has been proven safe and effective for them. Participants agreed with direct availability of test results to their health care practitioners but preferred to control access. Participants mostly ‘agreed’ that they would want all their relevant genetic factors to be analysed in one test. A minority (15.4%) either ‘agreed’ or ‘completely agreed’ towards worrying about their privacy if their results would be saved in a central medical record. Regarding financial costs, 119 participants (76.3%) ‘agreed’ or ‘completely agreed’ that they would be willing to take a pharmacogenomic test if this was partially reimbursed. This increased to 139 participants (89.1%) if the test was completely reimbursed (Table 2).

**Table 2:** Participant responses to pharmacogenomic test statements

	Completely disagree N(%)	Disagree N(%)	Neutral N(%)	Agree N(%)	Completely agree N(%)
Pharmacogenomic tests could help improve health care	0 (0.0%)	0 (0.0%)	20 (12.8%)	**88 (56.4%)**	48 (30.8%)
Pharmacogenomic tests could be an important aid during health care research	1 (0.6%)	1 (0.6%)	8 (5.1%)	**86 (55.1%)**	60 (38.5%)
I would feel more at ease taking a drug if pharmacogenomic tests have proved the drug is safe and effective for me	1 (0.6%)	4 (2.6%)	26 (16.7%)	**72 (46.2%)**	53 (34,0%)
I would improve my medication-adherence if pharmacogenomic tests have proved the drug is safe and effective for me	3 (1.9%)	26 (16.7%)	**54 (34.6%)**	43 (27.6%)	30 (19.2%)
I would worry about my privacy if my genetic data is saved in my central medical record	33 (21.2%)	**61 (39.1%)**	38 (24.4%)	18 (11.5.5%)	6 (3.8%)
I would prefer it if all my genetic factors are analysed in one test, making more information available, than having only the parts of my genetics analysed that could be relevant for my drugs	8 (5.1%)	16 (10.3%)	32 (20.5%)	**65 (41.7%)**	35 (22.4%)
I would like to limit the amount of health care practitioners that have access to my test results	8 (5.1%)	31 (19.9%)	42 (26.9%)	**47 (30.1%)**	28 (17.9%)
I would like a direct availability of my test results for my health care practitioners	4 (2.6%)	15 (9.6%)	29 (18.6%)	**88 (56.4%)**	20 (12.8%)
I would be willing to pay €100 for a pharmacogenomic test	11 (7.1%)	38 (24.4%)	**53 (34.0%)**	41 (26.3%)	11 (7.1%)
I would be willing to take a pharmacogenomic test if this was partially reimbursed	4 (2.6%)	5 (3.2%)	28 (17.9%)	**86 (55.1%)**	33 (21.2%)
I would be willing to take a pharmacogenomic test if this was completely reimbursed	1 (0.6%)	3 (1.9%)	13 (8.3%)	60 (38.5%)	**79 (50.6%)**

### Health care integration

Integration within health care was markedly preferred over direct-to-consumer (DTC) initiatives. Almost all participants trust their GP to initiate (92.9%) and sample tests, and to share results with (98.7%). Less than 1 out of 5 (16.7%) preferred a saliva test at home over a blood or saliva test performed by their physician (GP or specialist). Two thirds (63%) of participants would share their pharmacogenetic test results with their pharmacist as well, while DTC-providers were trusted with results by 6% only.

### Willingness to engage in pharmacogenomics research

**Fig. 3:**
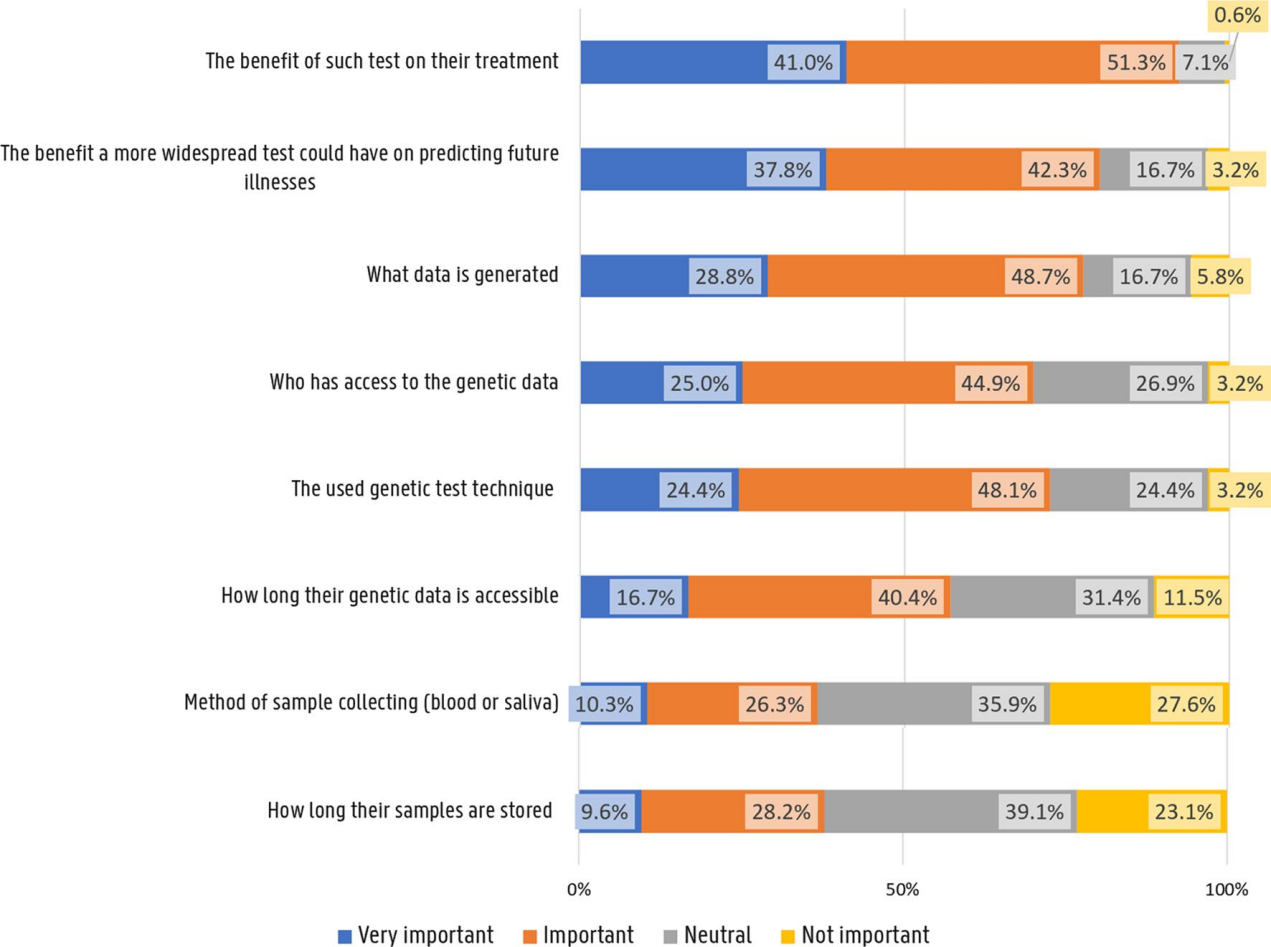
Importance of factors affecting participant’s willingness to engage in pharmacogenomic research

Overall, 71 participants (45.5%) were willing to participate in pharmacogenomic research involving drugs that are already on the market. Conversely, 28 participants (17.9%) would not be willing to participate and 57 participants (36.5%) were undecided. Figure 3 shows the answers to the questions regarding the importance of several factors on their decision to participate in research. Personal benefits in terms of more personalised treatment and prevention were most decisive for participants to engage in pharmacogenomics research while sampling methods and process were ranked the least important.

## Discussion

This was the first Flemish study on pharmacogenomics as a precision medicine tool to investigate public understanding and attitudes towards medication response and pharmacogenomic research. Almost half (45.5%) of our 156 participants were willing to participate in pharmacogenomics research with the main purpose of earlier disease treatment and prevention. Despite that less than a third (30.8%) had heard of pharmacogenomics or personalized medicine before, participants showed an overall positive attitude towards it, while recognizing the multifactorial nature of medication response including the importance of medication adherence.

While our sample was representative of a young, active population, half indicated to have experienced side effects or insufficient effect of a drug treatment before. This caused one third of participants to stop their drug due to side effects and almost half due to therapeutic failure. These findings are comparable to previous investigations of patient-reported side effects and medication non-adherence. Up to 40–50% of chronic diseases patients are estimated to be non-adherent, with side effects concerns being an important contributing factor to non-adherence [23]. For example, Cooper et al. reported that up 64% of asthmatic ICS users and 88% of asthmatic OCS users reported one or more side effect of their medication, and side effects concerns were associated with non-adherence [24]. Moreover, our study participants showed an interesting understanding of factors affecting their medication response with attaching more importance to their medication adherence, lifestyle, liver and kidney function than potential genetic effects. While this may not reflect a complete understanding of varying factors affecting medication response towards different drugs, it is in contrast to previous research showing limited understanding of medication research and development and limited knowledge of genetic variants involvement in medication response [25, 26]. The proportions of patients stopping their medication further emphasizes the importance of precision medicine as well as patient-provider communication, and approaches to reduce medication non-adherence.

Public willingness to participate in research is a cornerstone to translate research findings into clinical and general practice. Therefore, it is primordial to adequately inform and motivate the public about the expected results and the potential caveats of participating in scientific research. The percentage of participants ready to engage in pharmacogenomics research showed in our study (45.5%) is comparable to similar studies in the Netherlands and Japan (46.6% and 45.3%, respectively), perhaps showing a common trend among developed economies [19, 20]. Although this percentage may seem satisfactory, it shows that researchers should still make every effort to engage the undecided or reluctant proportions of society, to ensure a wider generalizability of pharmacogenomics research, essential for adequate interpretation and future applicability of the findings.

Participants indicated personal benefits in terms of more personalised treatment and prevention the most important for their decision to engage in pharmacogenomics research. They had strong opinions about who should perform pharmacogenetics tests and have access to its results with great preference towards integration within their health care over direct-to-consumer initiatives. They had a less outspoken opinion about sampling methods and processes.

Future pharmacogenomic studies and/or implementation initiatives could therefore focus more on communicating goals, interests and benefits to patients and participants and less to procedure and methodological details.

Overall, participants showed a positive attitude towards pharmacogenomics, strongly agreeing that pharmacogenomics could help improve health care and can be used as an aid during health care research. Furthermore, our results indicate that only 15.4% of our participants had concerns if their test results would be saved in a central medical file. This is in line to the previously mentioned study in the Netherlands, where 12.1% of their participants were concerned about the privacy of their genetic information [19] and considerably lower than a German survey where 36% of participants (adults with asthma or COPD) worried about privacy violations [27]. These findings are in contrast to previous suggestions that privacy could be a major barrier limiting the uptake of pharmacogenomic tests [14, 15, 17, 22, 28]. Integration within health care was markedly preferred over direct-to-consumer (DTC) initiatives within our study, with greatest confidence in their GP. However, it cannot be ruled out that limited privacy concerns could be in part due to limited interactions with the healthcare system in both our study or the Dutch survey, as opposed to the German study participants who had already undergone a pharmacogenomic test, and therefore could be better informed on the potential for privacy violation. Therefore, privacy concerns should still be addressed, especially as up to 94.2% participants were particularly concerned about the possible involvement of private companies or health insurance funds. Moreover, only a minority of participants were interested in DTC testing, potentially due to privacy concerns compared to tests conducted within the healthcare system. This is in line with earlier findings that participants trust their healthcare providers with their data more than commercial partners [29]. It could be argued as well that pharmacogenetic testing may not be suitable as a DTC service compared to other genomic tests (e.g.: ancestry testing). Pharmacogenetic testing, or tests predicting disease risk have a high potential for misinterpretation by the user, which could negatively affect their health status (for example: misunderstanding a drug to be dangerous due to a pharmacogenetic test result, and stopping its use). These types of tests could be either limited to the healthcare setting, or subject to a requirement for companies to request healthcare provider input for individual tests. Importantly, our participants mainly valued pharmacogenetics research to treat their disease earlier or, ideally, even prevent it. Early modification of future disease risk could be useful for many common illnesses, such as atherosclerosis, stroke, diabetes, cancer, dementia and depression [30]. The purpose of systems medicine research (including -omics markers such as genomics) is indeed to early identify, modify or prevent disease trajectories. Improved risk prediction could also foster non-pharmacological preventative strategies, such as regular health monitoring (e.g. cholesterol, organ function, premalignant changes) and lifestyle changes [30]. However, it is important that patients are educated on the limitation of predictive tests. Many common disorders are due to a combination of both genetic and environmental factors and the current polygenic risk scores are still limited [30]. Moreover, many complex diseases can only be understood by multi-omic studies where epigenomic and transcriptomic factors are taken into account [31, 32]. Taking only one aspect into consideration limits the sensitivity and specificity of (pharmaco) genomic tests [30, 33]. Therefore, these tests may only provide a probability for developing the disease in the future, a fact that should be emphasized in informed consents especially with more DTC tests available on the market [30, 33]. While interest was still low, up to 22.4% of our participants already had heard of DTC testing, showing a growing popularity and an important potential for over-ambitious or false promises, or misinterpretation of those tests.

Our study had several limitations. First, the survey was disseminated online only due to COVID-19 restrictions on public areas during the study period. This may have affected our ability to reach both elderly (1.3%) and/or less educated participants, underrepresenting important sectors of the population. Therefore, our sample represents a young, well-educated and active population without chronic conditions and their health literacy could have affected their good understanding of medication response, and trust and willingness to participate in pharmacogenomics research. Moreover, we relied on our participants prior knowledge of the complex subject of pharmacogenomics and precision medicine although only 30.8% had heard about the term before the survey. It should be taken into account that this limited knowledge of pharmacogenetics may have affected several of the participant answers during our study including the potential of pharmacogenetics to improve health outcomes. Nevertheless, recent trends in vaccine hesitancy may also show that an important percentage of reluctance towards healthcare interventions may stem from skepticism rather than inadequate education [34]. Moreover, there is an ongoing debate regarding the moral argument for privacy compared to the health value of collecting data to the improvement of both community and individual health [35]. Finally, we relied on our participants self-reported experience of side effects and medication efficacy, which could be subject to recall and confirmation bias. Some participants might have mistook delayed effect as lack of efficacy, or wrongly stopped a medication due to a side effect. Prior knowledge and health literacy could have affected these outcomes as well.

## Conclusion

In conclusion, citizens demonstrated a large interest in pharmacogenomics to personalize their therapy and modify future disease risk. Participants showed willingness to participate in pharmacogenomics research (45.5%), but indicated the need to be well informed about the research. A majority of participants believed pharmacogenomics could help doctors prescribe the right medications, with a minority showing privacy concerns when it is integrated within their health care. Overall, these results can be used to guide future pharmacogenomic implementation initiatives to pave the way for wide scale implementation within health care.

## Supplementary Information

**Additional file 1:** Survey questions.

**Additional file 2: **Analysis Dataset.

## Data Availability

All data generated or analyzed during this study are included in this published article and its supplementary information files. The dataset is included as a supplementary file. (Additional file 2).

## Abbreviations

DTC: Direct-to-consumer
ADRs: Adverse drug reactions
PGx: Pharmacogenomics
CPIC: Clinical pharmacogenomics implementation consortium
FDA: Food and drug administration
EMA: European medicines agency
ICS: Inhaled corticosteorids
OCS: Oral corticosteroids
COPD: Chronic obstructive pulmonary disease
