# Supplementary material for: Precision medicine from a citizen perspective: a survey of public attitudes towards pharmacogenomics in Flanders
Source: BMC Med Genomics. 2022 Sep 12;15(Suppl 3):193. doi: 10.1186/s12920-022-01308-7 (PMC9466314; doi:10.1186/s12920-022-01308-7)
Supplement: Supplementary file 1 — Additional file 1: Survey questions. [file 12920_2022_1308_MOESM1_ESM.docx]

**Survey Pharmacogenomics**

*Information note:* Thanks to scientific research, we know more and more about our genes. Genes contain the code that, for example, determines the colour of our eyes, but they can tell us much more. Within the field of pharmacogenomics we investigate if these hereditary pieces of code can also predict whether a medicine is safe or effective. Taking into account the differences of genetic background, the choice and dosage of a drug can be tailored to the patient. The aim is to pursue a safer and more effective treatment. Although this field has made great international progress and pharmacogenomic tests are becoming more affordable, research and implementation in Flemish clinical care is limited. To better understand the needs, expectations and preferences of the Flemish public, we would like to question your perspective through this questionnaire.

This survey can be completed anonymously and your answers will be analysed anonymously as well. In agreement with the General Data Protection Regulation (GDPR) (EU) 2016/679 of April 27, 2016, your privacy will be respected. If the results of this study will be published, your will be anonymity is insured. This study was approved by an independent Committee of Medical Ethics, tied to the University Hospital of Ghent and the University of Ghent, and will be executed following the guidelines of the ICH/GCP which was prepared in the statement of Helsinki to ensure the protection of individuals participating in clinical studies. Furthermore, a liability insurance was provided in accordance with the Belgian Experiments Act. (dd 7 mei 2004) (Allianz Global corporate & specialty, polis n° BEL000862). *Prof. dr. apr. Lies Lahousse*

□ I have read the information note above, fill this in voluntarily and give permission for my answers to be scientifically analysed and published.

1. What is your age?

- 18-30
- 31-50
- 51-70
- 70+

1. What is your sex?

- Male
- Female
- I’d rather not say

1. Wat is your education level?

- Elementary or primary degree
- High school degree
- College degree
- University degree
- Doctorate

1. How many drugs do you take daily?

- 0
- 1-5
- 5+

1. Have you ever experienced side-effects due to your drugs?

- Yes
- No
- Not that I know
- Not applicable

1. If you have ever experienced side-effects, was the drug treatment stopped?

- Yes
- No
- Not applicable

1. Have you ever taken a drug that appeared to not reduce your symptoms?

- Yes
- No
- Not that I know
- Not applicable

1. If you have taken a drug that didn’t appear to reduce your symptoms, was the drug treatment stopped?

- Yes
- No
- Not applicable

1. Please indicate how you believe that the following factors affect the safety or efficacy of drugs?

|  | No effect | Some effect | Large effect |
| --- | --- | --- | --- |
| My age |  |  |  |
| My weight |  |  |  |
| My gender |  |  |  |
| My lifestyle (e.g. smoking, alcohol consumption) |  |  |  |
| My medication-adherence |  |  |  |
| My genetic background |  |  |  |
| My kidney function |  |  |  |
| My liver function |  |  |  |

**Medication-adherence is the degree to which you take the drug as prescribed*

1. Order the following health care professionals in order of likelihood you would ask them about drug information? (first ranked = most likely)?

- Pharmacist
- General practicioner
- Specialist
- Nurse

1. Have you ever heard of pharmacogenomics or personalised medicine before the survey?

- Yes
- No
- I don’t know

1. Do you believe that your genetic background could help clinicians to prescribe you the right drugs?

- Yes
- No
- I don’t know

1. Do you believe your genetic background affects OTC drugs?

- Yes
- No
- I don’t know

1. To what extent do you agree with the following statements?

|  | Completely disagree | Disagree | Neutral | Agree | Completely agree |
| --- | --- | --- | --- | --- | --- |
| Pharmacogenomic tests could help improve health care. | o | o | o | o | o |
| Pharmacogenomic tests could be an important aid during health care research | o | o | o | o | o |
| I would feel more at ease taking a drug if pharmacogenomic tests have proved the drug is safe and effective for me | o | o | o | o | o |
| I would improve my medication- adherence if pharmacogenomic tests have proved the drug is safe and effective for me | o | o | o | o | o |
| I would worry about my privacy if my genetic data is saved in my central medical record. | o | o | o | o | o |
| I would prefer it if all my genetic factors are analysed in one test, making more information available, than having only the parts of my genetics analysed that could be relevant for my drugs. | o | o | o | o | o |
| I would like to limit the amount of health care practitioners that have access to my test results. | o | o | o | o | o |
| I would like a direct availability of my test results for my health care practitioners. | o | o | o | o | o |

1. As with other laboratory tests or medical procedures, there is a cost associated with pharmacogenetic testing. To what extent do you agree with the following statements?

|  | Helemaal oneens | Oneens | Neutraal | Mee eens | Helemaal mee eens |
| --- | --- | --- | --- | --- | --- |
| I would be willing to pay €100 for a pharmacogenomic test | o | o | o | o | o |
| I would be willing to take a pharmacogenomic test if this was partially refunded | o | o | o | o | o |
| I would be willing to take a pharmacogenomic test if this was completely refunded | o | o | o | o | o |

1. Rank the following clinical scenarios from highest priority to lowest priority for why you would take a pharmacogenomic test. (first ranked = highest priority)

- If the test can prove that a drug (in that dose) is safe for me
- If the test can prove that a cheaper alternative drug would be as efficient for me
- If the test can identify what disease(s) I will probably have in the future, so that the prevention can be started sooner
- If the test can prove that a drug (in that dose) is effective for me

1. Who should be able to initiate a pharmacogenomic test?

|  | Yes | No |
| --- | --- | --- |
| General practitioner | o | o |
| Specialist | o | o |
| Pharmacist | o | o |
| Yourself | o | o |
| Government (e.g. heel prick) | o | o |

1. Order the following health care professionals in order of likelihood you would ask them about pharmacogenomic information? (first ranked = most likely)?

- Pharmacist
- General practicioner
- Specialist
- Nurse

1. Have you heard of commercial direct-to-consumer (DTC) genetic tests through the internet or commercials?

- Yes
- No
- I don’t know

1. If yes, would you ever order such tests?

- Yes, I already have
- Yes, in the future
- No
- I don’t know
- Not applicable

1. Who should have acces to the genetic test results?

|  | Ja | Nee |
| --- | --- | --- |
| General practicioner (medical file) | o | o |
| Specialist (medical file) | o | o |
| Pharmacist (medicine file) | o | o |
| Yourself (medicine pasport) | o | o |
| Commercial provider (report) | o | o |

1. If you were invited to participate in research for pharmacogenetic test for existing drugs, would you participate?

- Yes
- No
- I don’t know

1. What information do you find important on your decision to participate?

|  | Not important | Neutral | Important | Very important |
| --- | --- | --- | --- | --- |
| Who has access to the genetic data | o | o | o | o |
| The used genetic test technique | o | o | o | o |
| How long the genetic data is accessible | o | o | o | o |
| How long the samples are stored | o | o | o | o |
| What data is generated | o | o | o | o |
| The benefit of such test on their treatment | o | o | o | o |
| The benefit a more widespread test could have on predicting future illnesses | o | o | o | o |
| Method of sample collecting (blood or saliva) | o | o | o | o |

1. Order in preference of where you would like a pharmacogenomic test to take place? (first ordered = highest preference)

- General practice (blood or saliva)
- Pharmacy (saliva)
- Specialists office (blood or saliva)
- At home (saliva)

1. Is there any other information you find important to know before taking pharmacogenomic tests or participating in research?
2. If you wish to be contacted by the University of Ghent in the future to participate in pharmacogenomic research, please leave your email address below:
